# A roadmap to ALS prevention: strategies and priorities

**DOI:** 10.1136/jnnp-2022-330473

**Published:** 2023-01-23

**Authors:** Michael Benatar, Stephen A Goutman, Kim A Staats, Eva L Feldman, Marc Weisskopf, Evelyn Talbott, Kuldip D Dave, Neil M Thakur, Ammar Al-Chalabi

**Affiliations:** 1 Department of Neurology, University of Miami Miller School of Medicine, Miami, FL, USA; 2 Neurology, University of Michigan, Ann Arbor, Michigan, USA; 3 Staats Life Sciences Consulting, Los Angeles, California, USA; 4 Department of Environmental Health, Harvard T.H. Chan School of Public Health, Boston, Massachusetts, USA; 5 Department of Epidemiology, University of Pittsburgh Graduate School of Public Health, Pittsburgh, Pennsylvania, USA; 6 ALS Association, Washington, District of Columbia, USA; 7 Department of Basic and Clinical Neuroscience, Maurice Wohl Clinical Neuroscience Institute, King's College London, London, UK

**Keywords:** ALS

## Introduction

Amyotrophic lateral sclerosis (ALS) is often considered a relatively rare disease, but risk estimates suggest that the lifetime risk of ALS is 1:263 for males and 1:417 for females by age 85.^1^ Therapeutic efforts to date have failed to meaningfully slow disease progression or prolong survival. This may, at least in part, reflect that treatment is typically initiated late in the disease process, when damage may already be too advanced to reverse. Indeed, some evidence suggests that earlier treatment may yield better outcomes.^2 3^ These observations prompt a reassessment of potential approaches for treating ALS, with a shift towards a proactive strategy for preventing ALS.^4^

Achieving the ambitious goal of preventing ALS requires a large body of knowledge, including an understanding of the causes and risk factors for ALS. It also requires identifying a window of opportunity to study those at risk for disease, methods for predicting when the clinical manifestations of ALS will emerge, and viable strategies to intervene by mitigating risk or treating the underlying biology of disease before clinically manifest ALS emerges. Significant progress has already been made in a small subset of the population, which is at markedly elevated genetic risk for ALS^4^ by harbouring highly penetrant ALS-causing gene mutations. When the cause of disease is known, for example, genetic cause, it is possible to identify and study presymptomatic gene mutation carriers.^5^ Moreover, biomarkers, notably, neurofilament light chain (NfL), have been identified that predict the risk of imminent phenoconversion,^6 7^ and experimental therapeutics that target the underlying cause of disease have begun to emerge.^8 9^

This confluence of factors has facilitated the launch of the first-ever ALS prevention trial (NCT04856982) of a genetic therapy, tofersen, in carriers of highly penetrant *SOD1* mutations, which are associated with rapidly progressive ALS.^10^ In this study, NfL levels are monitored monthly, and eligible presymptomatic people randomised to receive tofersen (an *SOD1* antisense oligonucleotide) or placebo when NfL levels rise above a predefined threshold. The goal of the trial is to delay, or possibly even prevent, the emergence of clinically manifest ALS.

Preventing ALS is substantially more challenging when the cause of disease is unknown. Although it is generally accepted that genetic background, cumulative environmental exposure and advancing age combine to cause ALS,^11 12^ less is known about the non-genetic factors that contribute to ALS. The magnitude of the challenge may seem overwhelming in the general population given its size (~350 million in the USA alone) and the low annual incidence of disease (~2 per 100 000 persons).^13^ However, several innovative strategies have yielded insights, including large-scale genetic studies,^14^ Mendelian randomisation studies, epidemiological analyses^15^ and epigenetic studies.^16^

Notwithstanding the challenges, it is possible to identify populations at greater risk for ALS than the general population, although at much lower risk than populations harbouring highly penetrant ALS-causing gene variants. Recognising at-risk populations yields opportunities to work towards disease prevention. Also, the risk of disease for individuals carrying disease-causing genetic variants may increase in the context of particular environmental exposures.^17^ Therefore, the appropriate prevention strategy for higher risk groups will vary based on multiple factors spanning population size, annual risk of disease, current knowledge base and the feasibility of the relevant research approach. Below, we identify some potential groups that could be prioritised for further prevention research.

## Higher-risk groups for study and targeting of prevention efforts

### People with genetic risk factors

Genetic risk factors for ALS broadly fall into three groups: (1) those that increase risk by a few percent, but because they are relatively common in the population, might represent a considerable risk in a person harbouring multiple such variants (eg, *UNC13A* single nucleotide variants); (2) those that increase risk more dramatically, by a few-fold (perhaps up to 300%), but are not sufficient to definitely cause disease (so-called reduced penetrance variants, eg, *ATXN2* intermediate repeat expansions) and (3) those that dramatically elevate risk and are frequently associated with familial disease (eg, rare variants in the *SOD1* gene). Most of these variants show age-dependent penetrance, meaning that the risk of developing ALS increases with age. The approach to studying presymptomatic disease to potentially prevent ALS has been well described for people carrying high-risk genetic variants.^5^ However, understanding presymptomatic ALS to contemplate preventing ALS is more challenging in the first two populations, that is, those with genetic variants that individually increase risk by up to about 300%. It would require identifying potential cases by widespread sequencing along with a willingness and ability to enrol and prospectively follow a cohort of individuals for an extended period of time, knowing many may never develop ALS, and with the ethical and psychological implications to participants of enrolling in such a study.

### People who have already developed frontotemporal dementia

In addition to, and partially overlapping with, populations at elevated genetic risk for ALS, are people with frontotemporal dementia (FTD), 5%–10% of whom also develop ALS.^18^ Since people with FTD are relatively easily identifiable, it is possible to study presymptomatic ALS and formulate strategies to prevent ALS in this population already suffering from another neurodegenerative disorder. Such a study would entail longitudinal follow-up of people with FTD, and tracking exposures (behaviours, lifestyles, habits, etc) to identify factors that might be targeted for intervention to prevent the emergence of ALS. This approach will require meaningful collaboration with the community studying and providing clinical care for people with FTD. Indeed, such studies are already ongoing, but they would benefit from the expertise of the ALS community.^19^ We recognise, of course, that it may already be too late to intervene on any risk factor responsible for triggering both FTD and ALS, but studying this population yields an opportunity to understand why some people with FTD go on to develop ALS while others do not.

### People with mild motor impairment

An until recently overlooked population at elevated risk for ALS, are individuals who have mild motor impairment (MMI).^4 20^ MMI represents a clinical syndrome characterised by motor symptoms physical signs on examination indicating upper or lower motor neuron (LMN) pathology, or electromyographic (EMG) abnormalities indicating an underlying LMN syndrome. These symptoms, signs and EMG abnormalities represent a clear departure from normal but are not of sufficient severity or distribution to definitely support a diagnosis of ALS. MMI is a clinical entity that is non-specific but may be prodromal to ALS; it emerged from the study of presymptomatic gene mutation carriers. MMI may also occur in most people who go on to develop ALS.^21 22^ Indeed, it may be possible to assemble and study a cohort of people with MMI, a fraction of whom will progress to develop ALS, but many of whom will not. Such a study could shed light on the presymptomatic phase of both genetic and non-genetic forms of ALS, and aid in developing strategies for preventing progression from MMI to ALS.

### Relatives of people with ALS or other or other neurological and psychiatric diseases

Biological relatives of people with ALS without a known family history of ALS have an eightfold increased risk of developing disease versus the general population.^23^ This is still a small risk in absolute terms but reflects shared risk factors. Biological relatives of people with FTD or other neurological and psychiatric diseases (eg, schizophrenia) may also be at elevated risk of ALS.^24^ There may be opportunities to study these populations to better understand the mediators of increased ALS risk. Mitigating these factors could represent progress towards preventing ALS. However, the magnitude of this challenge should not be underestimated since even an eightfold increased risk only translates into an incidence rate of 16 per 100 000 person-years follow-up, which is still very low.

### Veterans

Similar strategies and challenges apply to studying military veterans whose risk of ALS (4 per 100 000 person-years) is twice that of the general population.^25–28^ Again, however, insights into the mediators of this increased risk may yield opportunities to intervene more broadly with the goal of preventing some forms of ALS. Long-term population-based studies of military veterans may be a viable approach.

### People with exposure to environmental risks

Perhaps the most significant challenge relates to the urgent need to identify modifiable environmental exposures that increase ALS risk.^29^ Environmental risks are implicated in most individuals with non-genetic ALS,^12^ and even in people carrying a penetrant ALS genetic mutation.^17^ Exposures occur throughout the lifespan and across occupational,^30^ residential^31^ and avocational settings, making measurement of these exposures difficult. While progress has been made in delineating risks, such as pesticide exposure,^31 32^ lead exposure^33^ and physical activity,^34^ our understanding of the role of environmental factors in mediating ALS risk remains in its infancy. Certainly, it has not yet matured to an extent that would make it is possible to make specific recommendations (eg, by reducing exposures) to meaningfully reduce the future risk of ALS. However, experience from the transient increase in ALS incidence in Guam related to neurotoxin exposure, for example, beta-methylamino-L-alanine in the seed of cycad plants, illustrates the potential impact of environmental exposures and dramatic impact of successful mitigation efforts.^35^

The research priorities are clear. There remains an urgent need to identify environmental risks for ALS, and understand how these interact with genotype and advancing age. This will entail developing better tools for quantifying environmental exposures, which almost certainly vary and accumulate over time, together with registries of people with ALS that include detailed clinical and personal history data with meticulously banked biological samples. It will also require defining the critical periods when such exposures are most impactful in mediating disease risk. Importantly, we recognise the challenges of translating identified environmental risk factors for ALS into viable prevention strategies. Unlike pharmaceutical interventions, where the path from discovery to preventative intervention is clear, the level of evidence required for recommending behavioural changes, for example, to diet or physical activity, or broader changes to the environment, is unclear. Thus, the path to implementing such non-pharmacological interventions is as yet unchartered. However, there may be opportunities to consider developing pharmaceutical interventions, which reduce the consequences of environmental risks. For instance, numerous drugs effectively lower the harms or risk of disease from dietary exposures.^36^ This drug development strategy might also be applied to preventing ALS in appropriately high-risk groups for identified risk factors.

### Other strategies to guide prevention efforts

Identifying and studying people at reduced risk for ALS might yield insights into factors that protect against ALS. To the extent that delineating these factors illuminates the biology or mechanism of their protective effect, they might have implications for prevention efforts. For example, an evaluation of prescription drug use in administrative health claims from US Medicare beneficiaries identified 10 drugs significantly associated with lower ALS risk, including drugs for hypertension, diabetes and cardiovascular disease.^37^ Additionally, tamoxifen was related to lower ALS risk and testosterone to a higher risk in females. Reproducing these results and acquiring insights into the causal associations between drug use and ALS risk, will be critical to the prevention agenda.^38^

Moreover, studying ALS gene mutation carriers that survive to old age without developing clinically manifest disease and the rare group of patients described as *ALS Reversals*,^39 40^ might yield insights into potential protective factors that could be harnessed for therapy development. This potential is illustrated by analogy to the discovery of homozygous mutations for the null allele of C-C chemokine receptor type 5 (CCR-5), which conferred resistance to HIV infection,^41^ in turn providing a foundation for developing a new class of drugs for treating HIV.^42^

## Conclusion

Because of the limited capacity of the central nervous system for repair, early treatment of ALS is preferred, before there has been significant neuronal loss. Preventing ALS takes this idea to its logical conclusion, by stopping the neuronal loss before it manifests. To this end, we have identified a series of research priorities to facilitate ALS prevention (box 1). Despite the many challenges to achieve this goal, developing appropriate studies and cohorts will accelerate progress, making ALS prevention, at least in some people, achievable in the future. Our ultimate objective is to develop interventions that can prevent ALS in people. While efforts are underway to do this in a subset of highly penetrant *SOD1* mutation carriers and will need to be done for those carrying penetrant mutations in other genes, it has never been attempted on a larger scale. Success will require the ALS community to address both scientific and organisational challenges.

Box 1 **Research priorities to facilitate ALS prevention**

- Support the infrastructure needed to expand ongoing efforts, such as the Pre-symptomatic familial ALS (*Pre-fALS*) study, to study those at elevated genetic risk for ALS, including the potential interactions between genetic and environmental risk factors.
- Develop the infrastructure to ensure access to appropriate genetic counselling and testing for all ALS patients and their unaffected family members.
- Partner with the frontotemporal dementia and other neurodegenerative and neuropsychiatric communities to study and mitigate ALS risk among different populations at elevated risk for ALS.
- Establish new cohorts, or leverage existing population-based cohorts, to study both environmental risk factors for ALS, as well as mild motor impairment (MMI) as a clinical syndrome that is prodromal to ALS. These cohorts should include military veterans. Formal recognition of MMI as a diagnostic entity, for example, by assigning a specific International Classification of Disease code, would facilitate such studies.
- Develop improved tools for capturing and quantifying environmental exposures in a scientifically rigorous manner that is least burdensome to ALS patients. This can be accomplished through direct epidemiological methods, as well as other approaches including Mendelian randomisation,^14 34 43^ epigenetic studies and modelling of risk factors using for example, the multistep model of ALS.^17^
- Develop a framework for determining the level of evidence required to translate knowledge of an environmental risk factors for ALS, into a pharmaceutical, behavioural or ecological intervention.
- Expand efforts to study those at reduced risk for ALS, with the goal of identifying protective factors that could be harnessed for future therapeutic development.
